# The effect of a new maternity unit on maternal outcomes in rural Haiti: an interrupted time series study

**DOI:** 10.1186/s12884-021-04062-3

**Published:** 2021-09-04

**Authors:** Tonya MacDonald, Olès Dorcely, Joycelyne E. Ewusie, Elizabeth K. Darling, Sandra Moll, Lawrence Mbuagbaw

**Affiliations:** 1 Department of Health Research Methods, Evidence and Impact, McMaster University, Health Sciences Centre, 1280 Main Street West, Hamilton, ON L8N 4K1 Canada; 2 School of Midwifery, Laurentian University, Sudbury, ON Canada; 3 Centre Médical Béraca, La Pointe, Haiti; 4 School of Epidemiology and Public Health, University of Ottawa, Ottawa, ON Canada; 5 McMaster Midwifery Research Centre, McMaster University, Hamilton, ON Canada; 6 Department of Obstetrics and Gynecology, McMaster University, Hamilton, ON Canada; 7 School of Rehabilitation Science, McMaster University, Hamilton, ON Canada; 8 Centre for the Development of Best Practices in Health, Yaoundé Central Hospital, Yaoundé, Cameroon; 9 Biostatistics Unit/The Research Institute, St Joseph’s Healthcare-Hamilton, Hamilton, ON Canada; 10 Division of Epidemiology and Biostatistics, Department of Global Health, Stellenbosch University, Stellenbosch, South Africa

**Keywords:** Maternal mortality, Haiti, Interrupted time series, Undesirable outcomes, Caesarean births

## Abstract

**Background:**

In Haiti where there are high rates of maternal and neonatal mortality, efforts to reduce mortality and improve maternal newborn child health (MNCH) must be tracked and monitored to measure their success. At a rural Haitian hospital, local surveillance efforts allowed for the capture of MNCH indicators. In March 2018, a new stand-alone maternity unit was opened, with increased staff, personnel, and physical space. We aimed to determine if the new maternity unit brought about improvements in maternal and neonatal outcomes.

**Methods:**

We conducted an interrupted time series analysis using data collected between July 2016 and October 2019 including 20 months before the opening of the maternity unit and 20 months after. We examined maternal-neonatal outcomes such as physiological (vaginal) births, caesarean birth, postpartum hemorrhage (PPH), maternal deaths, stillbirths and undesirable outcomes (eclampsia, PPH, perineal laceration, postpartum infection, maternal death or stillbirth).

**Results:**

Immediately after the opening of the new maternity, the number of physiological births decreased by 7.0% (β = − 0.070; 95% CI: − 0.110 to − 0.029; *p* = 0.001) and there was an increase of 6.7% in caesarean births (β = 0.067; 95% CI: 0.026 to 0.107; *p* = 0.002). For all undesirable outcomes, preintervention there was an increasing trend of 1.8% (β = 0.018; 95% CI: 0.013 to 0.024; *p* < 0.001), an immediate 14.4% decrease after the intervention (β = − 0.144; 95% CI: − 0.255 to − 0.033; *p* = 0.012), and a decreasing trend of 1.8% through the postintervention period (β = − 0.018; 95% CI: − 0.026 to − 0.009; *p <* 0.001). No other significant level or trend changes were noted.

**Conclusions:**

The new maternity unit led to an upward trend in caesarean births yet an overall reduction in all undesirable maternal and neonatal outcomes. The new maternity unit at this rural Haitian hospital positively impacted and improved maternal and neonatal outcomes.

**Supplementary Information:**

The online version contains supplementary material available at 10.1186/s12884-021-04062-3.

## Background

There is renewed commitment toward global health and well-being through the United Nations’ new development agenda, the Sustainable Development Goals (SDGs). Regarding maternal and newborn child health (MNCH), the third SDG aims to reduce the global maternal mortality ratio to less than 70 per 100,000 live births by the year 2030 and to end preventable deaths of neonates [1]. Currently, on a global scale almost half of maternal deaths (45%) and over one-third (36%) of neonatal deaths take place within 24 h of birth [2]. Major maternal complications related to unsafe abortion, high blood pressure during pregnancy, birth complications, and severe bleeding and infection after birth [3] lead to maternal mortality; and these complications subsequently impact rates of stillbirth and neonatal death [4]. The highest rates of maternal and neonatal mortality are in low and middle-income countries (LMICs) [5].

The Republic of Haiti is a LMIC that shares the Caribbean Island of Hispaniola with the Dominican Republic. Based on 2015 World Health Organization (WHO) estimates, Haiti has the highest maternal mortality rates in the Americas [6, 7] with 488 maternal deaths per 100,000 live births in 2015 [7] and neonatal mortality of 26 deaths per 1000 live births [8]. In terms of access to comprehensive emergency obstetric and neonatal care, only 42% of births are attended by skilled health personnel, 39% of births take place in a health facility, and 38% of neonates have postnatal contact with a health provider within 2 days of birth [9].

The WHO defines two sets of life-saving interventions pertaining to the treatment of major obstetric and neonatal causes of mortality and morbidity [2]. Basic Emergency Obstetric and Neonatal Care (BEmONC) interventions are related to the administration of particular medications (e.g., uterotonics), and skills and resources for assisted vaginal birth, manual removal of placenta or retained products of conception and neonatal resuscitation. In addition to all of the components of BEmONC, Comprehensive Emergency Obstetric and Neonatal Care (CEmONC) is further defined by the ability to provide for caesarean births and blood transfusions [2].

According to Haiti’s 2019–2023 National Strategic Plan of Sexual and Reproductive Health, 39% of births in Haiti take place in a health facility of which 89% of these are institutions having CEmONC units and 11% with BEmONC units [6, 10]. In Haitian health facilities with CEmONC units, there is a 12% caesarean section rate [10]. On the same island, in the Dominican Republic over 95% of births occur in health care facilities, and 59% of live births are by caesarean section [11].

Efforts to increase access to comprehensive emergency obstetric and neonatal care are expected to reduce maternal and neonatal mortality, but these efforts must be tracked and monitored to measure their success [2, 12–14]. Haiti’s Ministère de la Santé Publique et de la Population (MSPP) lacks a comprehensive information system to track and map health services access and health outcomes, and consequently this has negatively impacted the healthcare system [6]. Despite the Haitian government’s comprehensive National Health Policy to reduce mortality and morbidity by building an accessible, efficient and universal health system [15], meager resources hamper equitable implementation of their “Health Master Plan” throughout the country [6]. As a result, the utilization, delivery, and accountability of essential MNCH, as well as sexual and reproductive health services, are problematic in Haiti, especially in remote and underserved areas of the country [16].

To monitor progress aimed at improving MNCH toward national and global health goals, Haiti’s MSPP currently requires all healthcare facilities with maternal-neonatal health services to monitor, track and report monthly on nine key outcomes from “the labour and birth room” including maternal mortality rate, stillbirth rate, and other outcomes such as utilization of prenatal care, intrapartum and postpartum complications, and utilization of intrapartum obstetric interventions [17]. In parts of Haiti where local surveillance efforts allow for the capture of MNCH indicators, these data can be used to measure progress towards both national MNCH goals and the global SDGs aimed at improving maternal and newborn well-being.

In order to improve MNCH outcomes in Haiti and ultimately reduce maternal mortality and neonatal mortality, local community healthcare facilities must; 1) continue to accurately track and report outcomes; 2) share findings in order to consider and effect potential changes that may improve outcomes; and 3) evaluate the effectiveness of interventions made to improve MNCH outcomes. Once a change has been put in place, an exploration of the effectiveness of the recent changes that were expected to improve MNCH outcomes, is needed. While interventions undertaken at the local community level should be monitored, appropriate choice of research design must also be considered. Experimental study designs such as Randomized Control Studies are expensive [18], logistically challenging and it would be unethical to randomize participants to a control group in which they were not offered the benefits of the best available care, i.e. the staff and facilities of the new maternity unit.

Within health system intervention research, a quasi-experimental research design called an Interrupted Time Series (ITS) study can be a feasible alternative to other designs. The ITS is a “multiple baseline” study design especially valuable for analysis of population-level health interventions [19] and holds the potential for a high degree of internal validity [20]. An ITS can be used to evaluate effectiveness of a health intervention at a specific point in time through a continuous sequence of observations of a specific outcome of interest, taken repeatedly over time and usually at equal intervals, to determine an underlying trend (also known as the counterfactual) that then becomes interrupted with the introduction of an intervention at a known and specific time point [19]. This study design allows for understanding of the effects of interventions (preintervention and postintervention), refinement of interventions and can be used to inform decisions about policies related to interventions [18]. The use of ITS methods can be rapidly implemented and provide flexibility of design, i.e. practical, cost and time-efficient study of one community with data availability through a central data repository or via mobile devices [18, 21]. Our study pertains to a local community healthcare facility in rural Haiti that sought an evaluation of their new maternity unit and had readily available data.

We aimed to determine if a new maternity unit at a rural Haitian hospital in March 2018 brought about improvements in maternal and neonatal outcomes. We hypothesized that maternal-neonatal outcomes would improve in the months after the opening of the new maternity unit.

## Methods

### Design

We used a quasi-experimental interrupted time series design. This design was employed for its feasibility and practicality and to inform understanding of the effects of Centre Médical Béraca’s (CMB) health system changes as an intervention, decisions about policies related to the intervention and future research.

### Setting

We collected data from the labour and birth and postnatal units (together called “la maternité”) at CMB. This is a not-for-profit primary healthcare, private hospital, providing services to patients, including to pregnant, labouring and postpartum women/persons 24 h per day and 7 days per week, located on the northwest coast of Haiti, near the city of La Pointe, and serving a population of over 700,000 [22]. The unit is staffed by an obstetrics (medical and nursing) team including two Staff Obstetrician/ Gynecologists, a Chief Registered Nurse, rotating Charge Nurses, a Nurse-Midwife, Nurses and Auxiliary Nurses, and rotating Obstetric Resident learners. We used data from “la maternité” for the period of July 2016 to October 2019.

Prior to March 2018, CMB’s “la maternité” was integrated within the main hospital, next to the only operating theatre, alongside the Departments of Pediatrics, and General Surgery. In March 2018, health system changes at CMB took place with the opening of a newly constructed and stand-alone maternity-postnatal unit, located in a new and separate building from the operating theatre. This new maternity led to an increased number of patient beds (from 10 to 28), and additional obstetric staff (from 1 to 2), nursing staff (from 8 to 16) and cleaning staff (from 4 to 8); and the introduction of patient-aides, security staff and maternity unit chief. In addition, there was increased physical space to include rooms designated for triage, labour, birth, and for post-operative patients and nursing staff, new public sinks and shared patient bathrooms, and video screens for community education.

### Variables

Maternity unit maternal-neonatal outcomes included in each monthly report consisted of prenatal care use, intrapartum interventions (e.g., Active Management of the Third Stage of Labour), intrapartum and postpartum complications (e.g., eclampsia, labour dystocia, hemorrhage, postpartum infection), maternal deaths, and stillbirths (e.g., with or without macerations).

The maternal variables were:

1. Physiological births: proportion of births by spontaneous vaginal birth, or vaginal births by induction of labour or with vacuum and/or forceps assistance;
2. Caesarean births: proportion of births by abdominal surgery through caesarean section;
3. Postpartum hemorrhage: proportion of births with blood loss in early postpartum until discharge from hospital, of greater than or equal to 500 ml;
4. Maternal deaths: proportion of births with maternal death during labour, in birth or before discharge from the hospital after giving birth.

The neonatal variable was:

5. Stillbirths: proportion of all fetal deaths late in pregnancy, resulting in stillborn infants with and without macerations.

An “undesirable outcomes” variable is study-defined and included:

6. All undesirable outcomes were combined as one variable to include eclampsia, PPH, perineal laceration, postpartum infection, maternal deaths or stillbirths.

### Data sources/measurement

Data points for this study were drawn from hospital-based records of the labour and birth and postpartum units. These data points were collected retrospectively from online CMB-prepared MSPP monthly reports from July 2016 to March 2018 (20 months preintervention) and then from March 2018 to November 2019 (20 months postintervention) for a total of 40 months of data. Monthly data were compiled after the last day of each month. Where CMB online data were missing, CMB-prepared MSPP monthly reports were obtained directly from CMB and used to complete missing data, where possible.

### Statistical methods and data analysis

Baseline characteristics of variables are reported as counts (%) pre- and postintervention. For each variable we calculated monthly proportions (e.g., number of maternal deaths per total births) for each of the 20 months preintervention and 20 months postintervention. We considered the beginning of the intervention to be March 2018 (the month the new maternity was opened). We used ordinary least squares (OLS) regression-based time series to analyze our data, with Newey-West standard errors to handle autocorrelation, and one lag. This method attempts to overcome and correct both heteroskedasticity (heterogeneity of variances) and autocorrelation in the error terms. The models included terms to evaluate the following variables: a constant to represent level of outcome at baseline before the intervention (β_0_), a term for linear trend before the intervention (β_1_), a term for change in level of the outcome after the intervention (β_2_), and a term for change in trend after the intervention (β_3_). The full model is reported in Additional file 1. We report Beta (β) coefficients, corresponding 95% Confidence Intervals and *P*-values. Negative β coefficients indicate reductions over time. Model fit was assessed using the F-test statistic. We plotted graphs of the outcomes over time to illustrate trends. Statistical significance was set at alpha < 0.05 for all analyses. Statistics were conducted using Stata Version 16 [23].

## Results

### Descriptive data

A total of 5848 births were included in this study during the 40-month study period (mean 146 births per month; standard deviation 45) with 2662 (45.5%) occurring prior to the opening of the new maternity unit and 3186 (54.5%) births taking place in the postintervention period. The distribution of outcomes in the pre- and postintervention periods is shown in Table 1.

**Table 1:** Rates of maternal and neonatal outcomes before and after opening of new maternity unit

Variables	Preintervention count (%) ***N*** = 2662 (20 months)	Postintervention count (%) ***N*** = 3186 (20 months)	Total (%) ***N*** = 5848 (40 months)
Physiological births	1873 (70.4)	2008 (63.0)	3881 (66.4)
Caesarean births	789 (29.6)	1178 (37.0)	1967 (33.6)
Eclampsia	52 (2.0)	50 (1.6)	102 (1.7)
Postpartum hemorrhage	2 (0.1)	14 (0.4)	16 (0.3)
Perineal laceration	428 (16.1)	590 (18.5)	1018 (17.4)
Postpartum infection	11 (0.4)	10 (0.3)	21 (0.4)
Maternal deaths	6 (0.2)	12 (0.4)	18 (0.3)
Stillbirths	196 (7.4)	180 (5.7)	376 6.4)
Undesirable outcomes	695 (26.1)	865 (27.2)	1551 (26.5)

### Outcomes

#### Physiological births

**Fig. 1:**
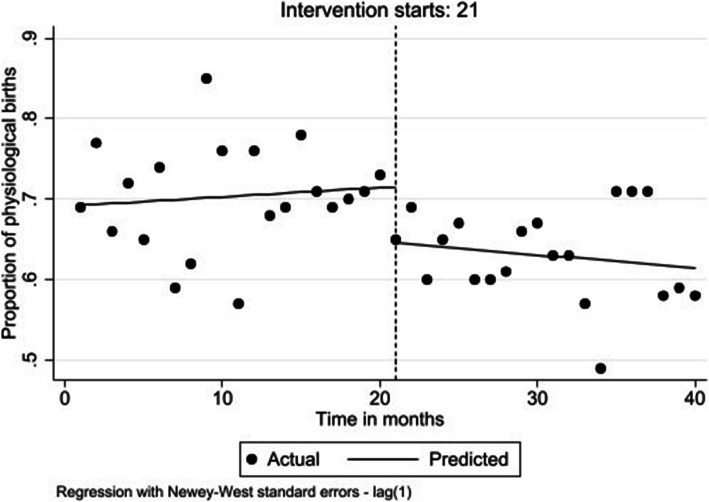
Level and trend change in proportion of Physiological births

Our results show that prior to the intervention, the trend in number of physiological births was stable (β = 0.001; 95% CI: − 0.001 to 0.004; *p* = 0.385). At the time of the intervention in March 2018, there was an immediate decrease in the number of all physiological births by 7.0% (β = − 0.070; 95% CI: − 0.110 to − 0.029; *p =* 0.001). In the postintervention period, this decreasing trend continued although it was not statistically significant (β = − 0.003; 95% CI: − 0.008 to 0.002; *p* = 0.264) (Fig. 1).

#### Caesarean births

**Fig. 2:**
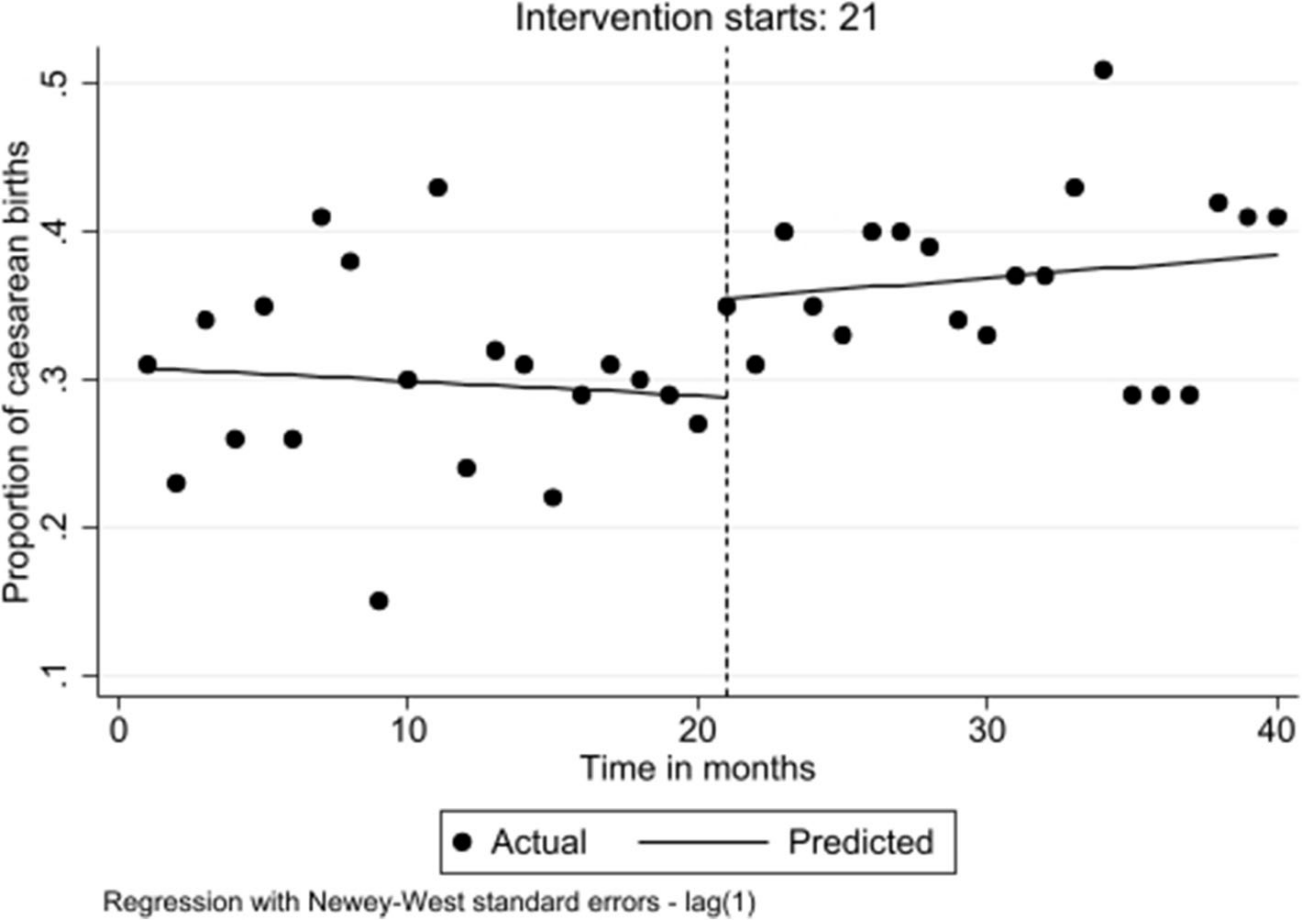
Level and trend change in proportion of Caesarean births

Before the intervention, the trend in caesarean births decreased marginally (β = − 0.001; 95% CI − 0.004 to 0.002; *p* = 0.449). There was an immediate and statistically significant increase of 6.7% with introduction of the intervention (β = 0.067; 95% CI: 0.026 to 0.107; *p* = 0.002). After the intervention through to the end of the postintervention period, the increasing trend continued although this was not of statistical significance (β = 0.003; 95% CI: − 0.002 to 0.007; *p* = 0.310) (Fig. 2).

#### Postpartum hemorrhage

**Fig. 3:**
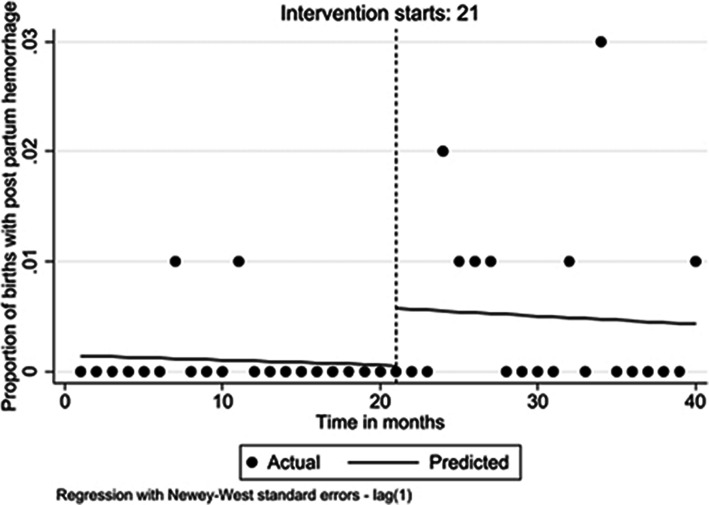
Level and trend change in proportion of women with Postpartum hemorrhage

Preintervention, the monthly cases of PPH were stable (β = 0.000; 95% CI 0.000 to 0.000; *p* = 0.533). But with introduction of the intervention in March 2018, there was an increase of 0.5% (β = 0.005; 95% CI: − 0.002 to 0.012; *p* = 0.150). There was no change in this trend in the postintervention period for PPH (β = 0.000; 95% CI: − 0.001 to 0.001; *p* = 0.922) (Fig. 3).

#### Maternal deaths

**Fig. 4:**
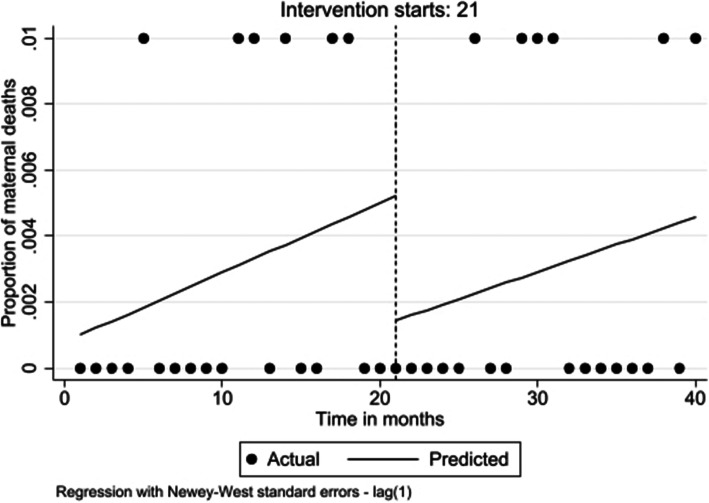
Level and trend change in proportion of Maternal deaths

The proportion of maternal deaths per month before the intervention, was stable (β = 0.000; 95% CI: 0.000 to 0.001; *p* = 0.220). At the time of the intervention, there was a decrease of 0.4% although this was not statistically significant (β = − 0.004; 95% CI: − 0.009 to 0.002; *p* = 0.181). During the postintervention period there was no change in maternal deaths per month (β = 0.000; 95% CI: 0.000 to 0.000; *p* = 0.835) (Fig. 4).

#### Stillbirths

**Fig. 5:**
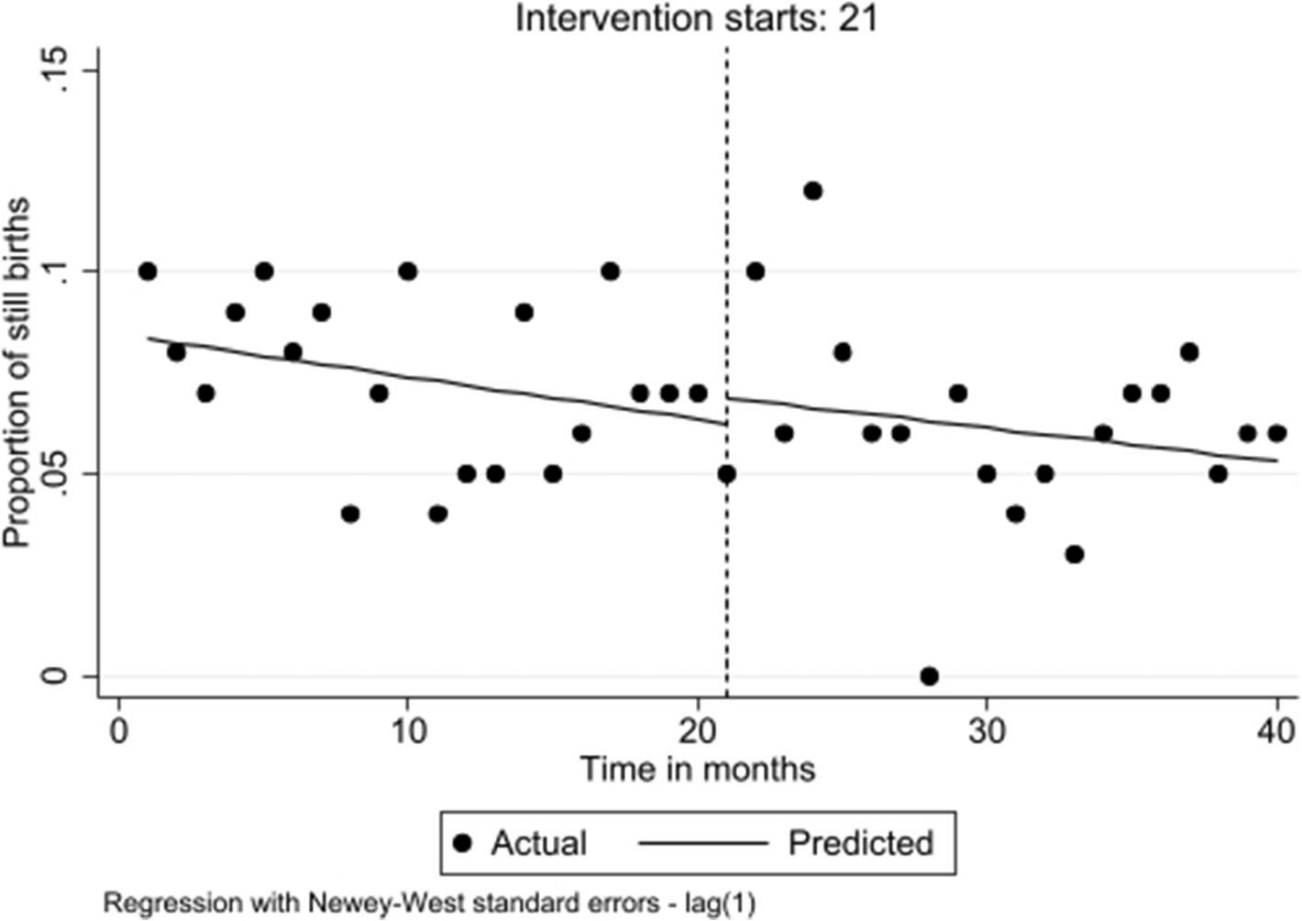
Level and trend change in proportion of Stillbirths

There was no significant change in the monthly rate of stillbirth in the preintervention period (β = − 0.001; 95% CI: − 0.002 to 0.000; *p* = 0.074). After the intervention, there was a small, non-significant increase of 0.6% (β = 0.006; 95% CI: − 0.022 to 0.035; *p* = 0.653) at the time of the intervention. The postintervention trend showed no increase in all stillbirths (β = 0.000; 95% CI: − 0.002 to 0.002; *p* = 0.826) (Fig. 5).

#### Undesirable outcomes

**Fig. 6:**
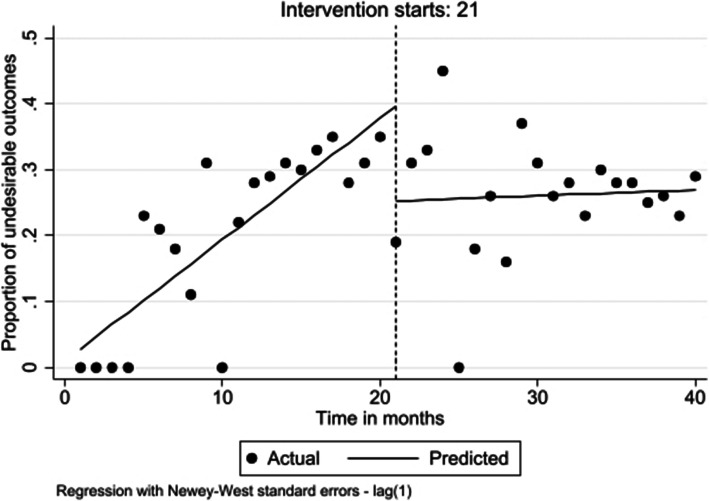
Level and trend change in proportion of undesirable outcomes

In the preintervention period, the proportion of undesirable outcomes per month showed an increasing trend of 1.8% (β = 0.018; 95% CI: 0.013 to 0.024; *p* < 0.001). At the time of the intervention there was 14.4% decrease of all undesirable outcomes (β = − 0.144; 95% CI: − 0.255 to − 0.033; *p* = 0.012). There was a decreasing trend of 1.8% through the postintervention period (β = − 0.018; 95% CI: − 0.026 to − 0.009; *p* < 0.001) (Fig. 6).

These outcomes are reported in Table 2.

**Table 2:** Outcomes predicting proportions of each maternal-neonatal variable to total monthly births

Outcome	Beta Coefficient	95% Confidence Interval	***P*** value
**Maternal variables**			
**Physiological births**^**a**^			
Preintervention baseline level *ß*_*0*_	0.693	0.649 to 0.736	< 0.001
Preintervention trend *ß*_*1*_	0.001	−0.001 to 0.004	0.385
Postintervention level change *ß*_*2*_	−0.070	−0.110 to − 0.029	0.001
Postintervention trend change *ß*_*3*_	−0.003	− 0.008 to 0.002	0.264
F(3,36) = 7.82; *p <* 0.001			
**Caesarean births**^**a**^			
Preintervention baseline level *ß*_*0*_	0.308	0.266 to 0.350	< 0.001
Preintervention trend *ß*_*1*_	−0.001	−0.004 to 0.002	0.449
Postintervention level change *ß*_*2*_	0.067	0.026 to 0.107	0.002
Postintervention trend change *ß*_*3*_	0.003	−0.002 to 0.007	0.310
F(3,36) = 7.17; *p <* 0.001			
**Postpartum hemorrhage**^**a**^			
Preintervention baseline level *ß*_*0*_	0.001	−0.001 to 0.004	0.222
Preintervention trend *ß*_*1*_	0.000	0.000 to 0.000	0.533
Postintervention level change *ß*_*2*_	0.005	−0.002 to 0.012	0.150
Postintervention trend change *ß*_*3*_	0.000	−0.001 to 0.001	0.922
F(3,36) = 1.74; *p* = 0.175			
**Maternal deaths**^**a**^			
Preintervention baseline level *ß*_*0*_	0.001	−0.002 to 0.004	0.507
Preintervention trend *ß*_*1*_	0.000	0.000 to 0.001	0.220
Postintervention level change *ß*_*2*_	−0.004	− 0.009 to 0.002	0.181
Postintervention trend change *ß*_*3*_	0.000	0.000 to 0.000	0.835
F(3,36) = 1.13; *p* = 0.349			
**Neonatal variables**			
**Stillbirths**^**a**^			
Preintervention baseline level *ß*_*0*_	0.083	0.070 to 0.097	< 0.001
Preintervention trend *ß*_*1*_	−0.001	−0.002 to 0.000	0.074
Postintervention level change *ß*_*2*_	0.006	−0.022 to 0.035	0.653
Postintervention trend change *ß*_*3*_	0.000	−0.002 to 0.002	0.826
F(3,36) = 3.40; *p* = 0.028			
**Undesirable outcomes**^**a**^			
Preintervention baseline level *ß*_*0*_	0.028	−0.043 to 0.099	0.433
Preintervention trend *ß*_*1*_	0.018	0.013 to 0.024	< 0.001
Postintervention level change *ß*_*2*_	−0.144	− 0.255 to − 0.033	0.012
Postintervention trend change *ß*_*3*_	− 0.018	− 0.026 to − 0.009	< 0.001
F(3,36) = 16.52; *p <* 0.001			

^a^Parameter as the proportion of the variable with respect to total monthly births

## Discussion

In this ITS study investigating the effect of opening a new maternity unit in rural Haiti, we found a reduction in physiological births, an increase in caesarean births and a reduction in undesirable outcomes.

The drop in physiological births immediately after opening the maternity is likely tied to the increase in caesarean births as there was a corresponding immediate and statistically significant increase in caesarean births. We first consider that during our study period, caesarean section rates at CMB ranged from 29.6% (preintervention) to 37% (postintervention). This range is higher than Haiti’s MSPP reported 12% section rate in health facilitates with CEmONC units [10]. It is also higher than findings in Boatin et al’s paper that show Haiti’s national average for caesarean section rate at 5.8%, with lowest rates among the poorest quintile at 1.6% and increasing with rising economic status to 17.9% for the richest quintile of women/birthing persons [24].

This range better aligns with rates of national averages of other countries from the region of the Americas that have both high national averages and high absolute wealth related inequalities in caesarean section rates [24]. Haiti’s national caesarean section rate of 5.8% barely meets the proposed range for optimal caesarean section rates of 5 to 20% - a range thought to capture and span both minimal desirable levels for emergency caesarean section and those representing overuse of elective caesarean section [24–26]. Yet CMB’s caesarean section rate is well-above Haiti’s average and has increased with the opening of the new maternity. In the community surrounding CMB, many women/birthing persons choose birth at home. Those who reach CMB with its CEmONC unit often arrive under circumstances that are clinically complex, urgent or grave in nature. Where life-saving measures are needed, caesarean birth is such an intervention. This may be a case of the new maternity providing the space and personnel to meet the local need for caesarean sections and attracting patients who may have a higher need for caesarean birth.

Second, we also consider optimal rates for medically necessary caesarean section. Determination of these is both challenging and controversial as to the true medical need for caesarean section at the population level, in order to achieve the best possible maternal and neonatal health outcomes [24]. At CMB, access to this life-saving intervention is possible because of local human resources and institutional infrastructure. However local medical staff, hospital administrators and policy makers must also consider possible inequities in its use, both underuse and overuse, and the short and long-term costs that unnecessary caesarean sections impose on their financially strained and burdened health system [27].

We found that PPH, maternal deaths and stillbirths were not impacted by the new maternity although these data were limited by small numbers of monthly cases.

However, the new maternity had an immediate and sustained impact by decreasing all undesirable outcomes. These findings indicate that the new maternity positively impacted and improved maternal and neonatal outcomes at CMB. This may be linked to the various evidence-based antenatal, intrapartum and postnatal interventions used at CMB that focus on the prevention of PPH, preeclampsia and eclampsia, maternal sepsis and obstructed labour, and lead to a reduction in morbidity and mortality [28]. In the new maternity these relate to space for patient triage, greater access to handwashing sinks, increased resources for electronic fetal surveillance, ongoing training and a dedicated space for neonatal resuscitation, and increased numbers of beds for longer lengths of stay. In LMICs a labouring person with prolonged or obstructed labour is more likely to die through complications such as a ruptured uterus, PPH, and maternal sepsis; or survive but suffer from morbidity related to issues of severe anemia, urinary incontinence and obstetric fistula, for example; or experience a stillbirth, neonatal death or neonatal infection [29]. A reduction in undesirable outcomes may also be related to cases of obstructed labour and treatment by caesarean section as appropriate and necessary (or assisted birth by forceps or vacuum) for management of obstructed labour, and saving maternal and neonatal lives [28].

Our findings provide some evidence in support of the overall effectiveness of improving the quality of care by creating new facilities in low-income countries.

Our study had some limitations. First, the retrospective collection of data from monthly reports and hospital records may introduce inaccuracies during compilation and transcription of data. We made every attempt to recover missing data, but lack of data may introduce bias in unforeseen ways, despite our attempts to consult multiple sources to obtain complete data. Second, our study had small counts for eclampsia, PPH, postpartum infection, maternal deaths and stillbirths, and thus limiting our precision. However, we combined all the undesirable outcomes into one variable to circumvent this issue. For neonatal outcomes, unfortunately monthly reports only track neonatal outcome data for: Stillbirths with macerations, and Stillbirths without macerations (which we combined these as all Stillbirths). Future research could include a retrospective chart review and would provide an elaboration of other neonatal outcomes. Third, other unmeasured factors such as changes in staff over time (e.g., new or more junior staff members) and changes in protocols or procedures on the unit (e.g., expectation to follow new protocol) may play a role in the observed trends. Future research might explore the impact of these personnel and organizational changes on patients, the obstetrics team and hospital staff, and provide further understanding of our findings. Fourth, the possibility that some other historical event or some other influence caused the observed effect in the time series limits our ability to make causal inferences [30]. Finally, the lack of a control group would suggest that our findings be treated as preliminary and interpreted with caution. A more robust ITS study design would control for this by use of a comparable control group [30].

The strengths of this study include a sufficient number of equally distributed data points (20) before and after the intervention, the use of patient-important and policy-relevant outcomes and adequate adjustment for autocorrelation.

Our study provided an inexpensive, rapidly implemented and practical opportunity for a rural Haitian community to examine the impact of opening a new maternity unit. The north-south collaboration provided shared learning and support for potential use of the ITS method in the future. This study provides the leaders of this hospital community with data about the effects of opening a new maternity unit in this rural Haitian community. Generalisability of these findings are limited to other rural Haitian communities where healthcare settings are contextually similar.

## Conclusions

Local surveillance efforts at a rural Haitian hospital have allowed for the capture of MNCH indicators, as one step toward the reduction of maternal and neonatal mortality and ultimately the improvement of maternal newborn child health. The opening of a new maternity unit increased caesarean births and reduced undesirable outcomes.

## Supplementary Information

**Additional file 1.** Interrupted time series model. Example of interrupted time series formula

## Data Availability

The datasets used and/or analysed during the current study are available from the corresponding author on reasonable request, and with approval and expressed consent from either CMB or Haiti’s Monitoring et Évaluation Surveillance Intégrée [17].

## Abbreviations

BEmONC: Basic Emergency Obstetric and Neonatal Care
β: Beta
CMB: Centre Médical Béraca
CEmONC: Comprehensive Emergency Obstetric and Neonatal Care
CI: Confidence interval
HiREB: Hamilton Integrated Research Ethics Board
ITS: Interrupted Time Series
LMICs: Low and middle-income countries
MNCH: Maternal and newborn child health
MSPP: Ministère de la Santé Publique et de la Population
PPH: Postpartum hemorrhage
*p*-value: Probability value
QI: Quality improvement
SD: Standard deviation
SDGs: Sustainable Development Goals
UEBH: Union Evangélique Baptistes d’Haïti
WHO: World Health Organization
