# Supplementary material for: The effect of a new maternity unit on maternal outcomes in rural Haiti: an interrupted time series study
Source: BMC Pregnancy Childbirth. 2021 Sep 4;21:601. doi: 10.1186/s12884-021-04062-3 (PMC8418005; doi:10.1186/s12884-021-04062-3)
Supplement: Supplementary file 1 — Additional file 1. Interrupted time series model. Example of interrupted time series formula [file 12884_2021_4062_MOESM1_ESM.docx]

**Additional file 1**

**Article title:** The effect of a new maternity unit on maternal and neonatal outcomes in rural Haiti: an interrupted time series study

**Author list:** Tonya MacDonald^1,2^, Olès Dorcely^3^, Joycelyne E Ewusie^1,4^, Elizabeth K Darling^1,5,6^, Sandra Moll^7^, Lawrence Mbuagbaw^1,8,9,10*^

**Interrupted time series model**

Where there is no comparison group and only one group under study, the standard ITSA regression model (distributed lag time series regression model) uses the following formula (adapted from Linden and Adams [1]:

**Y=** *ß_0_* **+** *ß_1_***Time_1_ +** *ß_2_***Intervention +** *ß_3_***Time_2_ + ε_t_**

Using the example of outcome variable maternal death (the proportion of births with maternal death):

**Y** is the aggregated outcome variable, maternal deaths occurring per month

*ß_0_* estimates the baseline level of maternal deaths (month 1)

*ß_1_* estimates the change in maternal deaths each month before the intervention (months 1-6)

**Time_1_** indicates the equally spaced time points that maternal deaths is measured (months 1-19 and months 20-40)

*ß_2_* estimates the change in monthly maternal deaths occurring immediately after the intervention (month 20)

**Intervention** is a dummy variable (0=before the intervention; 1=after the intervention)

*ß_3_* estimates the change in monthly maternal deaths after the intervention (months 20-40)

**Time_2_** is a variable indicating the number of months that have passed after the implementation of the intervention (0 for time before the intervention; 1-20 for time after the intervention)

**ε_t_** is the Error term; at time t, it represents the random variability not explained by the integrated time series model

**In ITS figures:**

*ß_0_*_:_  represents the starting level of the outcome of interest, and is the y-intercept (preintervention baseline level) (month 1)

*ß_1_*_:_ represents the slope or the trajectory of the outcome variable of interest until the introduction of the intervention (preintervention trend) (months 1-19)

*ß_2_*_:_ represents the change in the level of the outcome variable of interest during the phase following the intervention (postintervention level change) (month 20)

*ß_3_*_:_ represents the differences in slopes of the outcome variable between preintervention and postintervention (postintervention trend change) (months 20-40)

**References**

1. Linden A, Adams JL. Applying a propensity score-based weighting model to interrupted time series data: improving causal inference in programme evaluation. J Eval Clin Pract. 2011;17(6):1231-8.
